# A Personalized Health Monitoring System for Community-Dwelling Elderly People in Hong Kong: Design, Implementation, and Evaluation Study

**DOI:** 10.2196/19223

**Published:** 2020-09-30

**Authors:** Hailiang Wang, Yang Zhao, Lisha Yu, Jiaxing Liu, Inez Maria Zwetsloot, Javier Cabrera, Kwok-Leung Tsui

**Affiliations:** 1 Centre for Systems Informatics Engineering City University of Hong Kong Hong Kong China; 2 School of Design The Hong Kong Polytechnic University Hong Kong China; 3 School of Data Science City University of Hong Kong Hong Kong China; 4 Department of Systems Engineering and Engineering Management City University of Hong Kong Hong Kong China; 5 Department of Statistics and Biostatistics Rutgers University New Brunswick, NJ United States

**Keywords:** telehealth monitoring, personalized health, technology acceptance, digital biomarkers, digital phenotyping, wearables, falls detection, fitness tracker, sensors, elderly population

## Abstract

**Background:**

Telehealth is an effective means to assist existing health care systems, particularly for the current aging society. However, most extant telehealth systems employ individual data sources by offline data processing, which may not recognize health deterioration in a timely way.

**Objective:**

Our study objective was two-fold: to design and implement an integrated, personalized telehealth system on a community-based level; and to evaluate the system from the perspective of user acceptance.

**Methods:**

The system was designed to capture and record older adults’ health-related information (eg, daily activities, continuous vital signs, and gait behaviors) through multiple measuring tools. State-of-the-art data mining techniques can be integrated to detect statistically significant changes in daily records, based on which a decision support system could emit warnings to older adults, their family members, and their caregivers for appropriate interventions to prevent further health deterioration. A total of 45 older adults recruited from 3 elderly care centers in Hong Kong were instructed to use the system for 3 months. Exploratory data analysis was conducted to summarize the collected datasets. For system evaluation, we used a customized acceptance questionnaire to examine users’ attitudes, self-efficacy, perceived usefulness, perceived ease of use, and behavioral intention on the system.

**Results:**

A total of 179 follow-up sessions were conducted in the 3 elderly care centers. The results of exploratory data analysis showed some significant differences in the participants’ daily records and vital signs (eg, steps, body temperature, and systolic blood pressure) among the 3 centers. The participants perceived that using the system is a good idea (ie, attitude: mean 5.67, SD 1.06), comfortable (ie, self-efficacy: mean 4.92, SD 1.11), useful to improve their health (ie, perceived usefulness: mean 4.99, SD 0.91), and easy to use (ie, perceived ease of use: mean 4.99, SD 1.00). In general, the participants showed a positive intention to use the first version of our personalized telehealth system in their future health management (ie, behavioral intention: mean 4.45, SD 1.78).

**Conclusions:**

The proposed health monitoring system provides an example design for monitoring older adults’ health status based on multiple data sources, which can help develop reliable and accurate predictive analytics. The results can serve as a guideline for researchers and stakeholders (eg, policymakers, elderly care centers, and health care providers) who provide care for older adults through such a telehealth system.

## Introduction

In Hong Kong, residents aged 65 years old and above will account for 33.7% of the total population in 2066, compared to 17.0% in 2018 [1]. Aging reduces the physical and cognitive capacities of older adults and affects their ability to live independently or perform daily activities. Older adults are also susceptible to chronic diseases (eg, hypertension, diabetes, and dementia). For example, approximately 73% of Hong Kong residents aged 75 and above have hypertension [2] and nearly 1 out of 10 community-dwelling residents aged 70 or above has dementia. Managing such chronic diseases or their exacerbations is associated with close to 80% of health care budgets [3]. Another critical health issue for older adults is falling, which can result in decreased mobility level, fear of falling, and even death [4]. Approximately 18% of Hong Kong community-dwelling older adults experience falls. Among fallers, approximately 10% incur bone fractures [5] and around 32% experience soft tissue injuries [6]. These falls are associated with increases of up to HKD 552 million (US$70 million) in extra annual health care costs, approximately 30% of which can be reduced through an effective fall prevention program [7].

Recently, the Hong Kong government proposed a policy of “aging in place,” which encourages empowering older adults to remain in communities for long-term care services and to promote their well-being [8]. Such community-based services can ease the public financial burden as they are cheaper than public hospitals and can save costs that would be spent on misused health care resources (eg, unnecessary hospitalizations) [9]. Along with the shift from hospital care to community care, community-based health care systems are facing unprecedented challenges of limited capacity and resources to monitor older adults’ health continuously. Moreover, the caregivers in communities may lack professional knowledge and thus cannot detect health anomalies or suggest the next treatment for the elderly when needed. Therefore, innovative solutions for continuous monitoring of the health of the community-dwelling elderly population and linking with health care professionals are needed in the Hong Kong health system.

Owing to rapid developments in information technology, telehealth monitoring has provided cost-effective and timely access to quality care [10-12]. Telehealth monitoring systems utilize telecommunication technologies (eg, digital monitoring sensors) to capture and deliver health data (eg, vital signs) and services between patients and health care professionals. In particular, such a system provides a feasible solution to the increasing demand for long-term care support and monitoring to community-dwelling elderly individuals who may have difficulties in accessing health services [13-16]. A review of the current literature showed that telehealth systems have been assisting older adults in specific health-related areas such as chronic conditions [17-22], falls [23,24], and general wellness [25-28]. For example, Or and Tao [19] developed a patient-centered, tablet computer-based self-monitoring system to enable older adults with type 2 diabetes and hypertension to measure and monitor their blood glucose and blood pressure. Sparks and colleagues [22] proposed a decision support system that seeks to help community nurses monitor the well-being of their chronically ill patients, using an all-in-one station-based health monitoring device. Doty and colleagues [24] developed a wearable multimodal monitoring system designed for the real-life long-term monitoring of patients susceptible to falls. In addition to trial studies on telehealth monitoring systems, we also found some national telehealth programs that have been implemented, such as the Whole Systems Demonstrator program of the UK Department of Health [29], the Care Coordination/Home Telehealth program introduced by Veterans Health Administration in the United States [10], and the Home Monitoring of Chronic Disease in Aged Care program funded by the Australian government [16]. Significant benefits have been reported, such as a 19% reduction in numbers of hospital admissions [10] and 45% reduction in mortality rates [29].

However, as reported in previous studies [16,28,30,31], there are still some restrictions and challenges that could block the timely detection of health deterioration when implementing continuous monitoring systems, such as offline data processing and analysis, usage of individual smart devices, and single-parameter measurement. Motivated by these challenges, we aimed to integrate some state-of-the-art techniques into these monitoring systems, such as advanced biomedical signal analysis, statistical data analysis, predictive analytics, and decision support, which can help provide efficient health care services [32-34]. In addition, we sought to design a system that can utilize various smart devices to collect different health-related measurements for providing an efficient and accurate approach to raising health awareness in community monitoring [35,36].

## Methods

### System Design

#### Schematic Diagram

**Figure 1:**
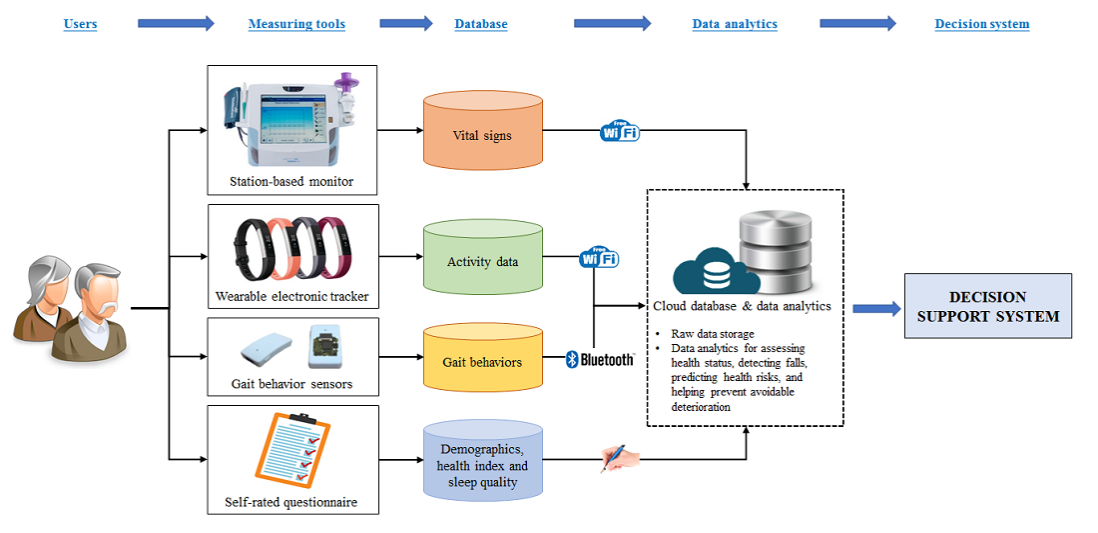
Schematic diagram of the proposed personalized health monitoring system.

Figure 1 shows the schematic diagram of our proposed personalized health monitoring system. The system captures and records older adults’ health-related information such as continuous vital signs and gait behaviors through various measuring tools, and will be integrated and analyzed using state-of-the-art data mining techniques. When any statistically significant changes in daily records are detected, the decision support system will emit warnings to older adults, their family members, and their caregivers, who can then take appropriate interventions to prevent further health deterioration. The details of the measuring tools integrated into the system are described below.

#### Vital Signs

Older adults’ vital signs are measured and recorded using an all-in-one station-based telemonitoring device (TMC, Telemedcare Systems Pty Ltd, Sydney, Australia). This device was selected based on a comprehensive and independent technology assessment process as described previously [9,16,22]. The vital signs that a TMC unit can measure include body temperature, systolic blood pressure (SBP), diastolic blood pressure (DBP), heart rate, and blood oxygen level (SpO_2_). The vital sign data can be electronically sent to a centralized database for quality control and diagnostic purposes.

#### Daily Activity

A commercial device (Fitbit-Alta, Fitbit Inc, USA) is integrated into our proposed system to record older adults’ steps and sleep data. All of these data will be synchronized and uploaded to the Fitbit cloud server for quality control and diagnoses.

#### Gait and Balance Sensor Signals

**Figure 2:**
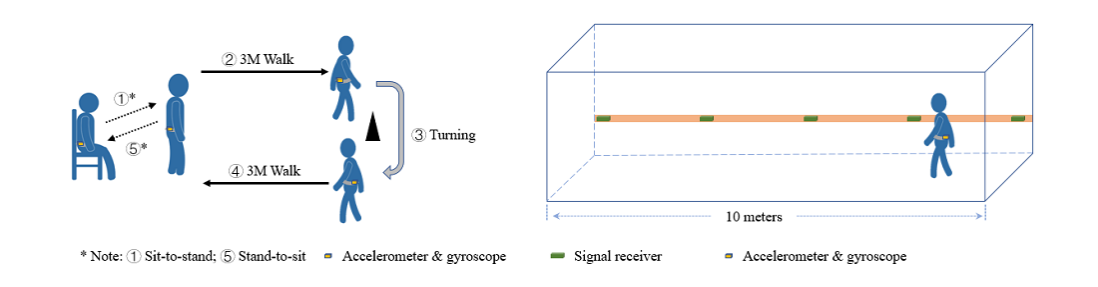
Illustrations of (left) the 3-meter timed up and go test and (right) the 10-meter straight walking test.

Older adults’ gait and balance status are measured using wearable sensors that are cost-effective with few constraints on monitoring movements [37-39]. Older adults need to put on a sensor (ie, accelerometer and gyroscope) before performing a 3-meter timed up and go (3M-TUG) test and a 10-meter straight walking (10M-SW) test (see Figure 2). The 3M-TUG test is a well-known clinical test of gait mobility [40] with high reliability [41]. During the 3M-TUG test, older adults need to stand up from a chair, walk 3 meters, turn around, walk back 3 meters, and sit down on the chair. The completion time of the 3M-TUG test is recorded as it is associated with impaired mobility and increased fall risks [40]. Gait speed is cited as the “sixth vital sign” [42] to reflect functional and physiological changes [43,44], and can further reflect fall risk [45]. Gait speed can be calculated from the 10M-SW test with participants walking 10 meters in a straight line. Signal data of gait behaviors are collected from the wearable sensor during the two gait tests. In addition, the Berg Balance Scale score is collected by registered physiotherapists [46,47] to identify older adults who are prone to falls and in need of preventive treatments [48,49].

#### Demographic Information, Sleep Quality, and Wellness Status

Customized questionnaires were used to collect older adults’ demographic information (eg, age, gender, and chronic disease history). Sleep quality was measured using the Pittsburgh Sleep Quality Index scale [50], one of the most commonly used clinical measures of sleep quality [51,52], with the score ranging from 0 to 21 (higher scores indicate worse sleep quality). Older adults were asked to self-report their daily wellness level, also called the Health Index, by rating on a 10-point scale from 1 (“feeling terrible”) to 10 (“feeling terrific”) [28].

### System Implementation

During the implementation phase, we collected raw data for system algorithm development and examined users’ acceptance of our proposed health monitoring system. A 3-month follow-up design was utilized in 3 centers that are part of a local nongovernment organization providing community services to the elderly [53]. Center A is a nursing home that provides 24-hour service to residents aged 60 or above and are mentally suitable for group living. Center B and Center C provide daycare services for residents aged 60 or above who live in the community.

#### Participants

Directors of the 3 centers first approached their center members, explained our study protocol, and invited older adults to participate. Based on the name lists from the directors, we recruited older adults who met all of the following inclusion criteria: (1) community-dwelling Hong Kong residents, (2) at least 60 years old, (3) willing to participate in the study, and (4) capable of cooperating in the assessment. Older adults with unstable or life-threatening illness were excluded. After completing all of the follow-up assessments, each participant was given a 50 HKD (US $6.50) supermarket coupon as a token of appreciation. The pilot study was approved by the Research Ethics Committee of City University of Hong Kong (reference number: 3-2-201803_02). All participants provided written informed consent before participating in the study.

#### Procedure

The implementation phase was scheduled from November 14, 2017 to February 13, 2018 in Center A; from December 17, 2017 to March 16, 2018 in Center B; and from March 1, 2018 to May 31, 2018 in Center C. We conducted follow ups every day during the 3-month period, excluding public holidays or special arrangements at the center (eg, special holiday leave in Center A; special training days in Center C). We ended up with a total of 58 follow ups for Center A, 63 follow ups for Center B, and 58 follow ups for Center C.

After obtaining participants’ consent forms, trained research assistants collected participants’ demographic and sleep quality information using questionnaires, and distributed each participant a Fitbit-Alta to wear 24 hours per day. The research assistants conducted the following operations in each follow-up visit, namely, every day during the 3-month period (except public holidays or special arrangements at the center).

First, the research assistants checked the battery of the Fitbit in use and, if needed, replaced it with a prepaired, fully charged Fitbit (each participant used two paired Fitbit devices during the pilot study). The research assistants then synchronized the Fitbit data with a tablet and uploaded it to the Fitbit server.

Second, the research assistants asked the participants to be seated in front of a TMC unit and guided each participant to use the TMC for measurements of vital signs. After all the measurements, the research assistants synchronized the vital sign data to the TMC server.

Third, the research assistants recorded the participants’ self-rated health status (ie, health index).

In addition, the research assistants measured participants’ body weight and performed a 3M-TUG test and a 10M-SW test once a week during the 3-month period (except for public holidays or special arrangements at the center).

### System Evaluation

Exploratory data analysis was performed to summarize the collected datasets. After the 3-month pilot study, we conducted a survey through distributing a questionnaire to evaluate users’ acceptance of the system. The participants were asked to rate their perceived acceptance of the system with respect to attitude (eg, “it is a wise idea to use this system”) [54], self-efficacy (eg, “it is comfortable to perform self-monitoring via the system”) [54], perceived usefulness (eg, “using this system for self-monitoring improves your health”) [55], perceived ease of use (eg, “learning to perform self-monitoring via the system is easy for you”) [55], and behavioral intention (eg, “you intend to perform self-monitoring using the system in the next 2 months”) [56], using a 7-point Likert-type scale, ranging from 1 (“very strongly disagree”) to 7 (“very strongly agree”).

## Results

**Figure 3:**
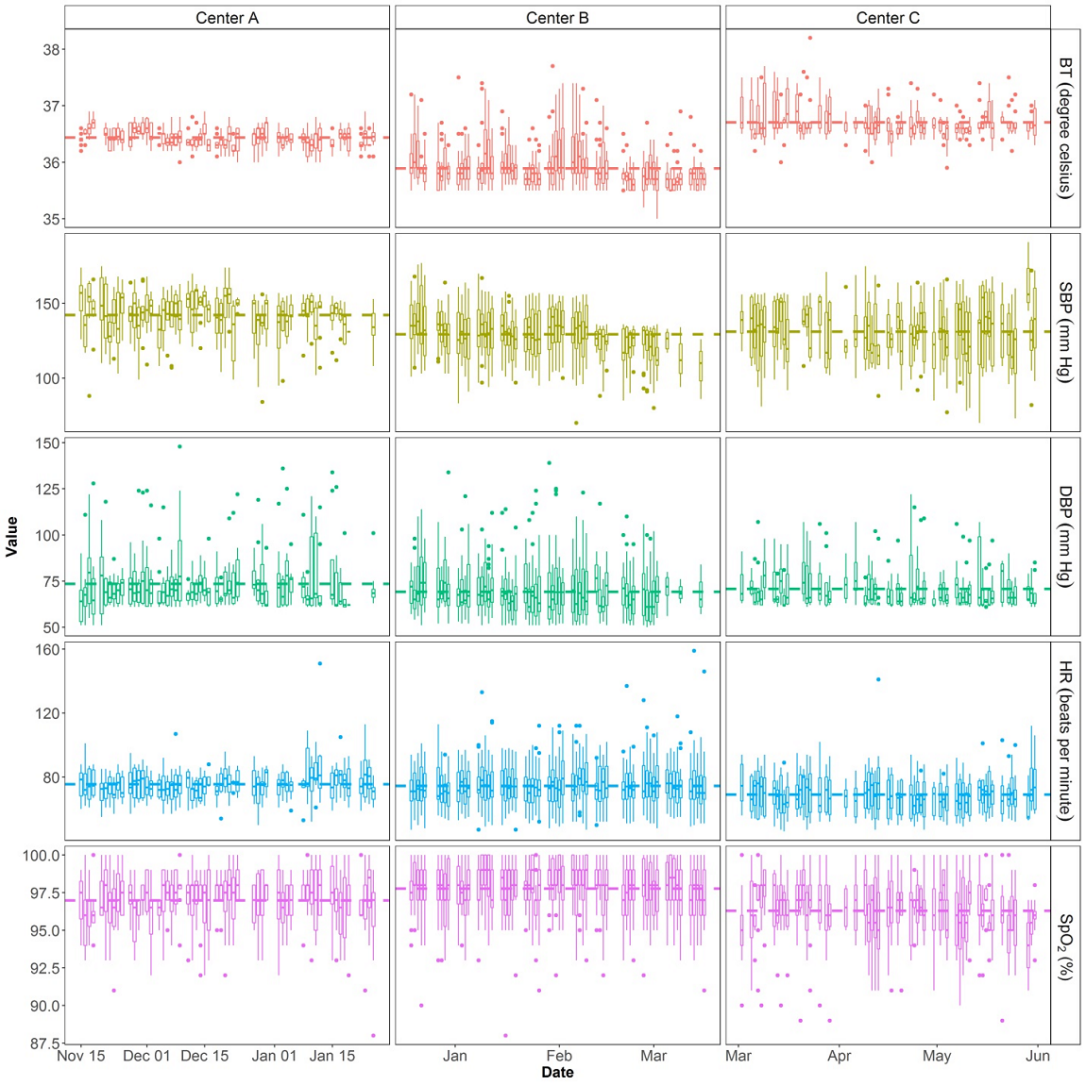
Vital signs of each participant from the 3 centers over time. The dashed lines represent mean values. BT: body temperature; DBP: diastolic blood pressure; SBP: systolic blood pressure; HR: heart rate; SpO_2_: blood oxygen level.

Table 1 presents the demographic information, daily activities, and vital signs of the 45 participants. Exploratory data analysis showed significant differences among group means in age (F_2,42_ =9.138, *P*=.001), steps (F_2,42_=33.9, *P*<.001), body temperature (F_2,42_=145.1, *P*<.001), and SBP (F_2,42_=4.417, *P*=.02). No other significant group differences were found. Figure 3 shows the longitudinal variations of the vital signs among the 3 centers.

**Table 1:** Demographics, daily activities, and vital signs of the 45 participants stratified by center.

Variable			Center A (n=10)	Center B (n=24)	Center C (n=11)
Age (years), mean (SD), range			88.7 (3.7), 82-94	76.3 (7.8), 61-91	81.4 (9.9), 71-106
**Gender, n (%)**					
	Female		8 (80)	21 (87)	4 (36.4)
	Male		2 (20)	3 (13)	7 (63.6)
**Chronic disease (self-reported), n (%)**					
	Hypertension		9 (90)	13 (54)	7 (58)
	Heart disease		2 (20)	3 (13)	2 (17)
	Stroke		0 (0)	1 (4)	3 (25)
	Diabetes mellitus		2 (20)	5 (21)	3 (25)
	Cancer		0 (0)	3 (13)	0 (0)
	High cholesterol		0 (0)	8 (33)	4 (33)
	Asthma		2 (20)	0 (0)	1 (8)
PSQI^a^, mean (SD)			7.80 (3.01)	7.42 (3.99)	7.00 (1.91)
Health index, mean (SD)			7.06 (1.72)	8.35 (1.24)	6.33 (1.60)
**Daily activities, mean (SD)**					
	Steps (number)		4751.8 (2481.7)	11817.5 (4089.1)	2751.5 (1718.5)
	Sleep (hours)		7.08 (1.77)	7.35 (1.60)	6.76 (2.14)
**Vital signs, mean (SD)**					
	Body temperature (℃)		36.45 (0.12)	35.89 (0.14)	36.72 (0.17)
	DBP^b^ (mmHg)		74.05 (7.58)	69.28 (8.37)	71.02 (4.28)
	SBP^c^ (mmHg)		142.20 (3.72)	130.10 (11.43)	130.63 (14.65)
	Heart rate (beats/minute)		74.99 (5.92)	74.19 (10.73)	70.64 (11.28)
	SpO_2_^d^ (%)		96.84 (1.62)	97.71 (1.29)	96.41 (1.90)

^a^PSQI: Pittsburgh Sleep Quality Index.

^b^DBP: diastolic blood pressure.

^c^SBP: systolic blood pressure.

^d^SpO_2_: blood oxygen level.

**Figure 4:**
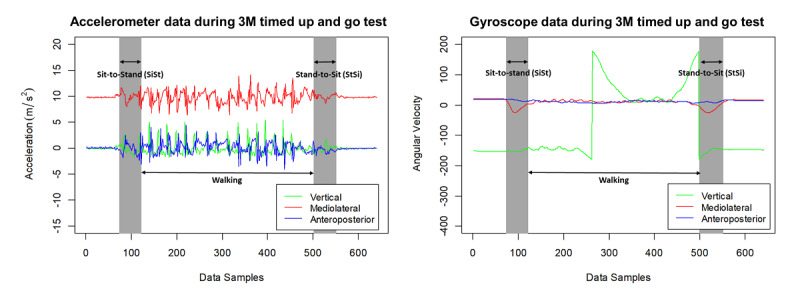
Example of segmented 3-meter (3M) timed up and go tasks using (left) accelerometer data and (right) gyroscope data.

Figure 4 shows an example of a segmented 3M-TUG task using accelerometer data and gyroscope data. Algorithms developed to segment the signal data into sit-to-stand, walking, and stand-to-sit are provided in our previous publication [57].

Overall, the participants strongly agreed that using the system is a good idea (mean 5.67, SD 1.06). The participants agreed that using the system is comfortable (mean 4.92, SD 1.11), useful to improve their health (mean 4.99, SD 0.91), and easy to use (mean 4.99, SD 1.00). In general, the participants showed a positive intention to use the first version of our personalized telehealth system in their future health management (mean 4.45, SD 1.78).

## Discussion

### Principal Findings

Innovative health care solutions such as telehealth are a possible solution to support community caregivers to meet the increasing health services demand. In this paper, we proposed our first version of an integrated, personalized telehealth monitoring system and demonstrated its implementation for Hong Kong community-dwelling older adults. We further evaluated its user acceptance after 3 months. This system can help communicate and manage the data collected from different sources, detect health anomalies, provide wellness prediction, signal alerts on health risks, and propose health improvement advice for reduction of adverse health outcomes.

For elderly individuals with chronic illnesses or at high risk of falls, timely detection of health anomalies is critical in health management. Any adverse conditions remaining untreated could result in a higher chance of hospitalization and longer recovery times [25]. Existing monitoring devices or systems mainly focus on the monitoring of vital signs, which has limitations for wellness prediction. The raw data from various sources can be of multidimensions, multiscales, and of varying precision. It is therefore important to develop tools and protocols for integrating and mining the personalized health-related data collected from various devices. Our proposed system integrates continuous measurements of vital signs, daily activities, and gait behaviors, allowing for better analytics and interpretation of disease progression and fall risks. Forecasting the wellness of the elderly based on identified personalized rules may be a useful indicator in early anomaly detection and potential treatment [25]. One of the key observations of our findings is that health-related data (eg, number of steps, body temperature, and SBP) showed high variations among the 3 test centers. For example, older adults who lived in Center A commonly exhibited a lack of physical activities, whereas those recruited from Center B exhibited a more active lifestyle with more physical activities. Such differences among various populations (eg, older adults at different care centers) may or may not affect the development of accurate predictive analytics. Thus, future studies with a large sample size are needed to validate the existence of population variations in health-related data and to further examine how such variations affect health predictions.

The collected data provide a rich resource to develop models and algorithms for smart personalized health management through risk assessment, and disease and harm prevention. For example, the vital sign data can be analyzed and modeled to identify biomarkers for anomalies in health status. Based on the automated risk stratification, decision support systems can be developed for patients themselves, their family members, or clinicians who can review patients’ health status and decide whether a health care service is needed.

### Implications and Future Work

Theoretically, our study offers an example of a system that provides multidimension, multiscale, and multiprecision data for elderly health monitoring. The integrated use of such data from multiple sources can offer more reliable information as compared with single-source data [28,58]. Based on the data collected from the proposed system, we have developed methods for incorporating data from multiple sources for predictive modeling (eg, wellness prediction for community-dwelling elderly people) [28,59]. For elderly health monitoring, it is important to combine continuous health monitoring data with discrete demographic/medical data. The variables consist of outcomes from sensing devices that output a continuous stream of sensor information that are related to various activities. Demographics/medical data consist of only a few variables collected discretely over a fixed time period. Naïve integration may result in situations where high-dimensional data dominate, and simple data summaries may cause significant loss of key information. New methodologies will be needed to determine the scale and dimensionality for best performance under various predictive models.

Following Chow [60], we propose to utilize different data fusion techniques for integrating heterogeneous data in terms of dimension, scale, and precision by means of statistical modeling. First, it is necessary to process and reconstruct collected data on a unified coordinate or reference grid. Here, we propose adopting a kernel-based smoothing method [61]. Given a measurement taken from a specific source at a specific time, the measurement will be filtered over a user-defined space-time domain through a kernel regression function. Details of this method are provided by Chow [60], who applied the method to the fusion of road traffic data. Second, data from different sources could be combined using the voting technique [62,63], which is essentially a weighted linear combination of information from different sources, in which the weights are defined according to the accuracy or creditability of the associated data sources as determined in advance. Moving forward, we will use heterogeneous longitudinal methods with a dynamic risk adjustment scheme for monitoring individual risk, such as the DySS, RA-CUSUM, and RA-EWMA methods [64,65]. In addition, we will identify major vital signs that affect health conditions based on health monitoring and lifestyle data, and then develop modeling strategies that will take the vital signs features as input for state-of-the-art machine learning algorithms such as boosting, support vector machine, random forest, ensemble modeling, and neural networks for wellness forecasting. Correlations between adverse health outcomes and multiple risk factors (eg, poor gait and balance) will be investigated based on the collected health monitoring data.

Practically, our system is likely to be of interest to policymakers, elderly care centers, and health care providers, particularly given the urgent need to increase the capability to care for the elderly as the burden shifts from hospital care. The proposed system involves linking health care providers with their patients without spending unnecessary time on less productive aspects of community activities such as avoidable driving to and from communities and on-site measurements of vital signs to assess health condition. The system has the potential to detect significant changes in health condition and to flag these changes as more caregiver attention is required to keep older adults out of hospitals. In addition, when hospitalization is needed, such a system may help to automate risk stratification of patients, which may facilitate hospital resources allocation. Thus, it can be beneficial to improving the quality of health care services provided, and ease the heavy burden on local health care systems. Moreover, end users may not initially accept and adopt a new technology after its introduction for a variety of reasons, and therefore will not experience the benefits. Our preliminary acceptance findings showed a relatively positive attitude for using our system (mean score=5.67) and slightly high levels of self-efficacy, perceived usefulness, perceived ease of use, and behavioral intention (with mean scores of 4-5). One possible reason could be that the participants may not have fully perceived the potential benefits of our system as the utilized system in the present study did not include any prediction algorithms for health management and timely communications between the system and the participants. Based on previous studies, there could be some other factors that significantly affect user acceptance of health information technology, such as social influence (eg, family/friends’ opinions) [66,67], facilitating conditions (eg, organizational and technical infrastructure) [68], and technology anxiety (eg, anxiety in using technology) [69].

To help implement our system into practice, we will perform further longitudinal acceptance modeling studies on the full version of our system to focus on the factors that affect older adults’ and health care professionals’ acceptance [70]. Following that, targeted strategies (eg, community-based technology support services and training workshops) will be promoted to improve user acceptance on our smart system. In the long run, our proposed research is expected to develop effective ways to reduce the growing elderly care burden on health care systems.

### Limitations

Missing data is a common problem encountered in most health care–related studies. Monitoring data quality in the presence of missing data is required, because the accuracy and reliability of measurements may be impaired when nonmedical experts perform the measurements [16]. One limitation of this study is the lack of data quality monitoring during implementation. Based on the data collected, we developed a data quality monitoring method to signal issues with the accuracy of the collected data quickly [71], which could be beneficial for further studies. Moreover, our study was based on a 3-month design and cannot evaluate the influences of season/time of the year on data variation. Future studies, in particular longitudinal studies of more than 6 months, are recommended to consider the evaluation of the effects of season/time of year.

## Abbreviations

3M-TUG: 3-meter timed up and go
10M-SW: 10-meter straight walking
DBP: diastolic blood pressure
SBP: systolic blood pressure
SpO_2_: blood oxygen level
